# Effectiveness of oncology nurse navigator on the incidence of postoperative pulmonary complications in gastric cancer patients undergoing radical gastrectomy

**DOI:** 10.1186/s12912-023-01291-z

**Published:** 2023-06-16

**Authors:** Yamin Yan, Peili Jin, Zhenghong Yu, Zhaoqing Tang, Jingjing Lu, Yan Hu, Yuxia Zhang

**Affiliations:** 1 Nursing Department, Zhongshan Hospital, Fudan University, NO.180, Fenglin Road, Xuhui District, Shanghai, 200032 China; 2 General Surgery Department, Zhongshan Hospital, Fudan University, Shanghai, 200032 China

**Keywords:** Gastric cancer, Radical gastrectomy, Oncology nurse navigator, Postoperative pulmonary complications

## Abstract

**Background:**

Management of postoperative pulmonary complications (PPCs) can be challenging in gastric cancer patients undergoing radical gastrectomy and is always associated with poor prognosis. Even though oncology nurse navigator (ONN) provide effective and critical individualized care to patients, little is known about their impact on the occurrence of PPCs in gastric cancer patients. This study aimed to determine whether ONN decreases the incidence of PPCs in gastric cancer patients.

**Methods:**

This was a retrospective review in which data for gastric cancer patients at one centre was evaluated before and after an ONN hired. An ONN was introduced to patients at their initial visit to manage pulmonary complications throughout treatment. The research was conducted from 1 August 2020 to 31 January 2022. The study participants were divided into the non-ONN group (from 1 August 2020 to 31 January 2021) and the ONN group (from 1 August 2021 to 31 January 2022). The incidence and severity of PPCs between the groups were then compared.

**Results:**

ONN significantly decreased the incidence of PPCs (15.0% vs. 9.8%) (OR = 2.532(95% CI: 1.087–3.378, P = 0.045)), but there was no significant difference in the components of PPCs including pleural effusion, atelectasis, respiratory infection, and pneumothorax. The severity of PPCs was also significantly higher in the non-ONN group (p = 0.020). No significant statistical difference was observed for the major pulmonary complications (grade ≥ 3) between the two groups (p = 0.286).

**Conclusions:**

Role of ONN significantly decrease the incidence of PPCs in gastric cancer patients undergoing radical gastrectomy.

## Background

Gastric cancer (GC) is the third leading cause of cancer-related death, implicated for almost 800,000 deaths every year [1]. Radical gastrectomy is widely considered the optimal therapy for GC. Postoperative pulmonary complications (PPCs) occur in 18.1% of gastric cancer patients [2]. PPCs are the major causes of poor prognosis pulmonary resection patients, and can increase intensive care unit (ICU) admission and prolong the length of hospital stay [3]. In recent years, deliberate efforts have been made to decrease the incidence of PPCs. Unfortunately, the management of these complications is still challenging. The current postoperative management involves monitoring patient behaviors, lifestyle adjustment, and coordinated multidisciplinary care over the perioperative period.

Although numerous methods for reducing PPCs have been proposed, the contribution of oncology nurse navigators (ONNs) in reducing PPCs has not been reported. In 2017, the Oncology Nursing Society defined ONN as “a professional RN with oncology-specific clinical knowledge who offers individualized assistance to patients, families, and caregivers, participating in overcoming the healthcare system barriers”. Regarding cancer, an ONN attends to patients’ needs (education, economic, medical, and psychosocial care, etc.) and provides critical information required for decision making in all phases of the cancer continuum [4].

Numerous ONNs and oncology studies in different centers are currently available. For example, in Turkey, the nurse navigation program significantly promoted participation behaviors and improved health-related beliefs concerning colorectal cancer screening [5]. Another study performed in Chicago and Texas showed that enhanced patient navigator assistance improves health-related quality of life among colorectal cancer survivors [6]. In breast cancer patients, the navigation program eased the treatment process and care coordination and ensured patients received high quality care [7]. Also, ONN significantly decreased the 30-day heart failure readmission rate and increased education and follow-up among heart failure patients [8]. An IBD-centered gastroenterology clinic revealed that incorporating a nurse navigator improved patient satisfaction, increased research participation, and decreased no-show rates [9].

The highlighted evidence shows that managing PPCs can reduce the length of hospital stay and mortality rates in cancer patients, which was the focus of this study. This study aimed to evaluate the impact of navigation programs on the incidence and severity of PPCs in gastric cancer patients undergoing radical gastrectomy. We hypothesized that navigation intervention significantly lowers the incidence and severity of PPCs.

## Materials and methods

### Subjects and study design

This was a retrospective study conducted in adult GC patients who underwent radical gastrectomy in GC center of Zhongshan Hospital, Fudan University. Records for GC patients seeking treatment at our center between August 2020 and January 2022. The ONN group comprised patients who received navigation care from August 2021 to January 2022, whereas the non-ONN group comprised patients who underwent the existing care from August 2020 to January 2021.

Subjects were enrolled using cluster sampling. The inclusion criteria were: (I) adult GC patients; (II) patients underwent elective radical gastrectomy; and (III) patients provided written informed consent. The exclusion criteria were: (I) patients with a diagnosis of gastric stromal tumor; (II) patients with cognitive impairment, unable to complete the training requirements; (III) patients with baseline demographics data or PPCs data missing or (IV) Patients who received radical gastrectomy during the training period (February 2021 to July 2021).

### Role of ONN

The ONN received extensive training before the commencement of the postoperative care of GC patients who received radical gastrectomy.

**Fig. 1:**
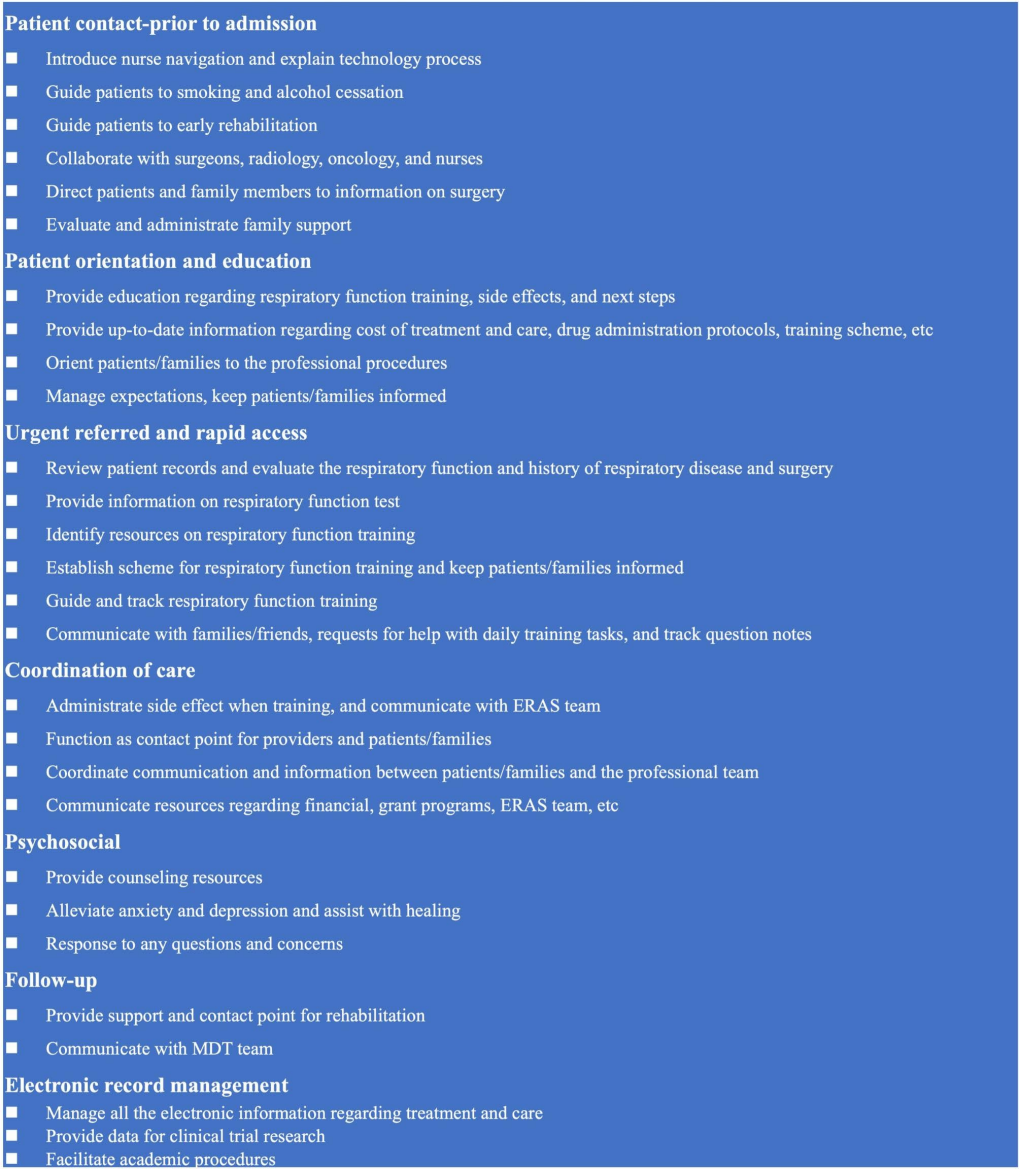
Interventions for managing PPCs in gastric cancer patients after radical gastrectomy

The interventions for managing PPCs are summarized in Fig. 1. The ONN focused on patient contact and education regarding respiratory training before admission. During the hospital stay, ONN oriented and educated the patients and advised on urgent referrals and urgent access, and coordinated clinical care and, psychosocial support. After discharge, electronic data over the follow-up period was analyzed.

### Outcome

The primary outcome assessed among the GC patients after radical gastrectomy was the incidence of PPCs within the hospital stay period. The PPCs were defined according to the European Society of Anesthesiology (ESA) and the European Society of Intensive Care Medicine (ESICM) guidelines. PPCs included respiratory infection, respiratory failure, pleural effusion, atelectasis, pneumothorax, bronchospasm, and aspiration pneumonitis [10]. Additional outcomes included the severity score and incidence of major pulmonary complications (grade ≥ 3).

### Data collection

Collection instrument in this study was a self-made questionnaire with the research variables. The items formulated through panel discussion and consultation with five GC specialist nurses and two GC specialist surgeons. The validated questionnaire revealed an adequate internal consistency (Cronbach’s alpha = 0.88), and the content validity index was 0.91.

Data in the electronic database included age, gender, type of surgery (laparoscopy or laparotomy), length of the hospital stay, and ICU admission was collected. The patients’ disease history, inspection report, laboratory blood test report, and temperature chart were assessed to ascertain the accuracy of the reported PPCs. All the data was exported to an electronic file for analysis.

### Statistical analysis

Continuous normally distributed variables were presented as mean ± standard deviation (SD). Median (interquartile range) was used for the non-normally distributed data, whereas categorical variables were presented as numbers and percentages. The median was used in place of missing data.

Cronbach’s alpha and content validity index were calculated for internal consistency and content validity of the questionnaire. Differences between the two groups for categorical variables were analyzed using the chi-square test, whereas the *t-*test or Mann-Whitney U test was used for continuous variables. The difference in the incidence and severity of PPCs (including composites of PPCs) between the two groups were analyzed using the chi-square or Fisher exact tests. The correlation between the ONN program and the incidences of PPCs was evaluated using logistic regression analysis.

The data were analyzed using the SPSS software, V. 26.0 (SPSS Inc., IBM Corp., Chicago, IL, USA). Statistical significance was set at P < 0.05.

## Results

### Sociodemographic and clinical characteristics of the study participants

A total of 893 GC patients were enrolled in this study, in which 432 patients received treatment in the hospital before hiring nurse navigator. Of these, 297(68.8%) were male, whereas 135(31.2%) were female. A total of 461 study participants comprising 323(70.1%) males and 138(29.9%) females, were cared for bynurse navigator from the day of reporting in the hospital. There were no significant differences in age, gender, smoking history, critical GC, surgery type, surgery time and postoperative hospital stay between the two groups. However, the rate of laparoscopic surgery was higher in the ONN group than in the non-ONN group (27.8% vs. 13.0%, p < 0.001). Before admission, the radiotherapy/chemotherapy rate was lower in the ONN group than in the non-ONN group (6.1% vs. 9.7%, p = 0.043) (Table 1).

**Table 1:** Demographics and clinical characteristics of the study participants

Variables	Non-ONN group (n = 432)	ONN group (n = 461)	t/χ^2^	P value
Age (year)	62.65$\pm$10.62	63.22$\pm$11.66	-0.776	0.444
Gender (%)			0.182	0.670
Male	297(68.8)	323(70.1)		
Female	135(31.2)	138(29.9)		
Smoking status			2.166	0.337
Never smoked	181(41.9)	201(43.6)		
former smoker	156(36.1)	146(31.7)		
Active smoker	95(22.0)	114(24.7)		
Family history of GC			4.647	0.031
Yes	58(13.4)	41(8.9)		
No	374(86.6)	420(91.1)		
History of pulmonary disease				
COPD	42(9.7)	34(7.4)	1.578	0.231
Asthma	16(3.7)	22(4.8)	0.625	0.508
Pulmonary infection	28(6.5)	30(6.5)	0.000	1.000
Others	16(3.7)	13(2.8)	0.554	0.572
Laparoscopic surgery			29.872	< 0.001
Yes	56(13.0)	128(27.8)		
No	376(87.0)	333(72.2)		
Critical GC			0.003	0.959
Yes	38(8.8)	41(8.9)		
No	394(91.2)	420(91.1)		
Radiotherapy/chemotherapy before admission			4.109	0.043
Yes	42(9.7)	28(6.1)		
No	390(90.3)	433(93.9)		
Scope of surgery			7.076	0.070
total gastrectomy	191(44.2)	175(38.0)		
distal gastrectomy	189(43.8)	238(51.6)		
proximal gastrectomy	25(5.8)	29(6.3)		
others	27(6.3)	19(4.1)		
Anastomotic methods			52.952	< 0.001
Bills I Bills II	111(25.7) 41(9.5)	99(21.5) 16(3.5)		
Bills II + Braun	43(10.0)	122(26.5)		
R-Y	204(47.2)	177(38.4)		
Others	33(7.6)	47(10.2)		
Surgery time(min)	175.24$\pm$68.02	180.60$\pm$83.14	-0.925	0.355
Postoperative hospital stay	8.47$\pm$3.22	8.10$\pm$3.15	1.744	0.082

### Incidence of PPCs

PPCs occurred in 65 of 432 (15.0%) and 45 of 461 (9.8%) patients in the non-ONN and ONN groups, respectively (p = 0.016). The incidences of PPCs, including pleural effusion (11.3% vs. 8.5%, p = 0.149), atelectasis (7.2% vs. 6.1%, p = 0.508), respiratory infection (2.3% vs. 1.1%, p = 0.195), and pneumothorax (0.2% vs. 0.9%, p = 0.375) were comparable between the two groups. Respiratory failure, bronchospasm, or aspiration pneumonitis were not observed among patients in either group (Table 2). Logistic regression analysis revealed a significant difference in PPCs between the two groups showed (OR = 2.532(95%CI: 1.087–3.378, P = 0.045)). Further analyses revealed that anastomotic methods and late-stage GC increase the risk of developing PPCs (Table 3).

**Table 2:** The incidence of PPCs in the two study groups

Variables	Non-ONN group(n = 432)	ONN group (n = 461)	χ^2^	P-value
PPC	65(15.0)	45(9.8)	5.767	0.016
pleural effusion	49(11.3)	39(8.5)	2.086	0.149
atelectasis	31(7.2)	28(6.1)	0.439	0.508
respiratory infection	10(2.3)	5(1.1)	2.044	0.195
pneumothorax	1(0.2)	4(0.9)	1.621	0.375
respiratory failure	0	0	--	--
bronchospasm	0	0	--	--
aspiration pneumonitis	0	0	--	--
Number of PPCs			6.385	0.089
0	367(85.0)	416(90.2)		
1	22(5.1)	17(3.7)		
2	39(9.0)	24(5.2)		
≥ 3	4(0.9)	4(0.9)		

**Table 3:** The correlation between nurse navigators’ care and the development of PPCs

Variables	S.E.	Exp(B)	95% Confidence Interval		P-value
			Lower	Upper	
Age	0.010	0.992	0.973	1.012	0.444
Sex	0.348	1.136	0.575	2.247	0.713
Smoking	0.321	0.787	0.419	1.476	0.455
Family history of GC	0.351	0.917	0.461	1.822	0.804
Laparoscopic surgery	0.333	0.763	0.397	1.466	0.417
Anastomotic methods					< 0.001
Bills I	0.565	0.751	0.248	2.271	0.612
Bills II	0.494	1.258	0.478	3.313	0.642
Bills II + Braun	0.324	0.299	0.158	0.564	< 0.001
R-Y	0.434	0.328	0.140	0.769	0.010
Radiotherapy/chemotherapy before admission	0.361	1.057	0.521	2.146	0.878
Critical GC	0.277	0.198	0.115	0.341	< 0.001
Navigation	0.224	2.532	1.087	3.378	0.045

### Severity of PPCs

**Fig. 2:**
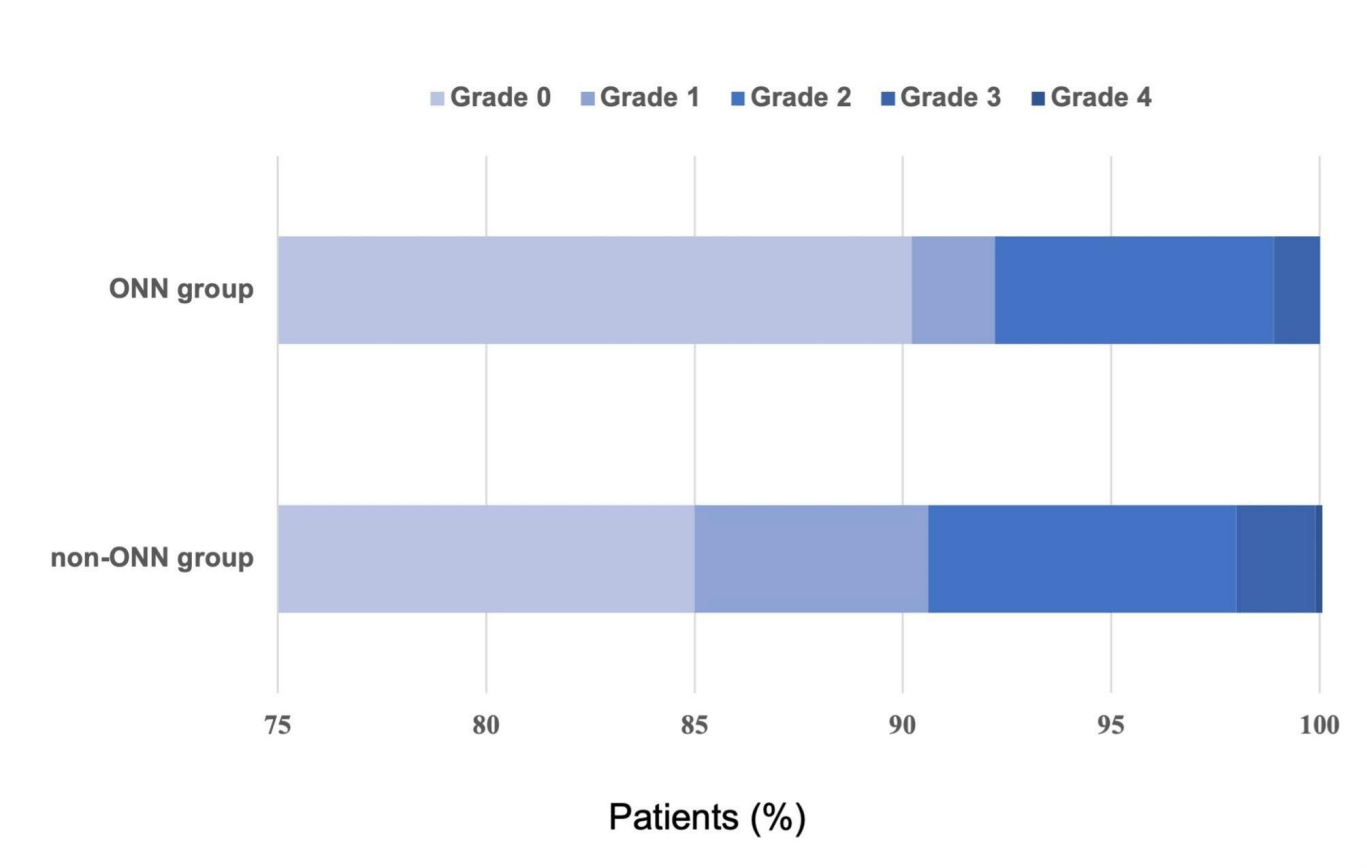
Severity score of PPCs between the two groups (Grade 0: no symptoms or signals for PPCs; Grades 1 to 4: successively worse forms of complications; Grade 5: death before hospital discharge)

There were significantly more severe PPCs in the non-ONN group than in the ONN group (p = 0.020) (Table 4; Fig. 2). Although the incidence of major pulmonary complications (grade ≥ 3) was higher in the non-ONN group than in the ONN group (2.1% vs. 1.1%), the differences were not significant (p = 0.286).

**Table 4:** Severity score of PPCs

Variables	Non-ONN group(n = 432)	ONN group (n = 461)	χ^2^	P-value
PPCs severity score			10.635	0.020
Grade 0	367(85.0)	416(90.2)		
Grade 1	24(5.6)	9(2.0)		
Grade 2	32(7.4)	31(6.7)		
Grade 3	8(1.9)	5(1.1)		
Grade 4	1(0.2)	0		
Grade 5	0	0		
PPCs grade ≥ 2	41(9.5)	36(7.8)	0.800	0.405
PPCs grade ≥ 3	9(2.1)	5(1.1)	1.442	0.286

## Discussion

In this study, we investigated the impact of ONN on the clinical outcome of GC patients after radical gastrectomy. PPCs were the primary outcomes assessed. We found that incorporating ONN in the care and treatment program significantly lowered the incidence and severity of PPCs among GC patients. However, ONN had no effect on the development of major pulmonary complications (grade$\ge$3), and components of PPCs among GC patients.

Hospitals and nurses are increasingly motivated to develop creative programs that improve the quality of clinical care. In surgical patients, PPCs management represents an important challenge in this field. PPCs are the leading cause of poor surgical outcomes, adversely influence surgical mortality, and significantly increases hospital stay and costs [11]. Recent evidence shows that the majority of patients undergoing abdominal surgery is pre-frail or frail [12, 13], and frail patients have multi-organ problems, associated with higher rates of adverse outcomes [14]. Functional frailty, such as respiratory muscle frailty, is an independent prognostic risk factor for PPCs [15]. However, there is no standard approach for managing PPCs in GC patients after radical gastrectomy. In recent years, the demand for ONN for cancer patients continues to increase [16]. Meanwhile, ONN improves the quality of care and treatment of mainly critical patients [17]. To the best of our knowledge, this is the first study reporting the beneficial effect of ONN, particularly in managing PPCs outcomes, as evidenced by the lower incidence and severity score of pulmonary complications.

The impact of navigation interventions is inconclusive. For instance, in his study, Green et al. revealed that navigation did not increase colonoscopy completion after a positive screening test within six months [18]. In contrast, Wang et al. reported that navigation breast oncology screening enhanced patients’ knowledge of disease and treatment compliance [19]. A related systematic review revealed comparable findings that navigation program shortens the time between screening, diagnosis, first consultation, and first treatment of cancer patients [17]. In the present study, we found that the overall incidence of PPCs among GC patients under the nurse navigation program was 12.3% (110/893), significantly lower than previously reported [20, 21]. Meanwhile, 65 of 432 (15.0%) vs. 45 in 461 (9.8%) patients in the non-ONN and ONN groups, respectively (p = 0.016). In the present study, pleural effusion was higher in the non-ONN (11.3%) than that in the ONN group (8.5%), consistent with a previous report [22]. No respiratory failure, bronchospasm, and aspiration pneumonitis occurred. The severity of PPCs was lowered in the ONN group than in the non-ONN group. Taken together, our findings showed that ONN-guided training programs can improve the treatment outcome of patients with terminal illnesses. This is consistent with a recent study that ERAS-based respiratory function training in older patients can prevent PPCs after abdominal surgery [23].

Studies have shown that severity of PPCs most likely stems from multiple reasons, such as complexity of surgery, difficulty coughing and expectorating, trauma stress, microvascular permeability, plasma colloid osmotic pressure (COP) [24, 25]. Furthermore, research has confirmed that intraoperative COP based goal-directed fluid therapy can reduce PPCs of grade 2 and higher severity [25]. In our study, complications analysis showed that 9 patients (2.1%) had PPCs of grade 3 or worse in non-ONN group, while 5 patients (1.1%) in ONN group. Although no statistical difference found between the two groups (p = 0.286), major pulmonary complications (grade ≥ 3) were numerically higher in the non-ONN group, potentially indicating the positive effects achieved by ONN. One possible explanation reason for lack of statistically significant difference is that the small sample size was insufficiently powered to detect a difference. Further large-scale clinical trials are still needed to verify the effect.

Regarding strength, this is the first study on the impact of ONN on the treatment out of GC after radical gastrectomy. On limitation, given that this was a retrospective study, we had no control over patient recruitment and data collection. Some variables, such as complexity of surgery, intraoperative blood loss, may affect the incidence of PPCs were not analyzed. Also, the recruitment of patients was non-randomized, given that the study participants were recruited from a single center. We adjusted all the confounding factors in the analysis to minimize the bias. Despite the promising result, large sample randomized controlled, multi-center clinical trials and physiological tests are still needed to verify the impact of ONN on severity of PPCs.

## Conclusions

In conclusion, ONN can potentially improve the treatment outcome of terminal diseases by reducing the incidence and severity of PPCs, but there was no evidence of its influence on the occurrence of major pulmonary complications (grade ≥ 3).

## Data Availability

The datasets generated and/or analyzed during the current study are not publicly available due to ownership by the Department of General Surgery, Zhongshan Hospital, Fudan University, Shanghai, China, but are available from the corresponding author on reasonable request.

## Abbreviations

GC: gastric cancer
PPCs: postoperative pulmonary complications
ICU: intensive care unit
ONNs: oncology nurse navigators
ESA: European Society of Anesthesiology
ESICM: European Society of Intensive Care Medicine
COP: colloid osmotic pressure
