# Correction: Effectiveness of oncology nurse navigator on the incidence of postoperative pulmonary complications in gastric cancer patients undergoing radical gastrectomy

**DOI:** 10.1186/s12912-023-01399-2

**Published:** 2023-07-28

**Authors:** Yamin Yan, Peili Jin, Zhenghong Yu, Zhaoqing Tang, Jingjing Lu, Yan Hu, Yuxia Zhang

**Affiliations:** 1 Nursing Department, Zhongshan Hospital, Fudan University, NO.180, Fenglin Road, Xuhui District, Shanghai, 200032 China; 2 General Surgery Department, Zhongshan Hospital, Fudan University, Shanghai, 200032 China

**Correction to: BMC Nursing (2023) 22:208**

10.1186/s12912-023-01291-z

Following publication of the original article [1], the authors reported that “grade” was missing from “grade≥” in the 6 parts of the article content. The correct sentences are given below and the changes have been highlighted in **bold typeface**.

## Abstract

No significant statistical difference was observed for the major pulmonary complications (**grade** ≥ 3) between the two groups (p = 0.286).

## Outcome

Additional outcomes included the severity score and incidence of major pulmonary complications (**grade** ≥ 3).

## Severity of PPCs

Although the incidence of major pulmonary complications (**grade** ≥ 3) was higher in the non-ONN group than in the ONN group (2.1% vs. 1.1%), the differences were not significant (p = 0.286).

## Discussion

Although no statistical difference found between the two groups (p = 0.286), major pulmonary complications (**grade** ≥ 3) were numerically higher in the non-ONN group, potentially indicating the positive effects achieved by ONN.

## Conclusions

In conclusion, ONN can potentially improve the treatment outcome of terminal diseases by reducing the incidence and severity of PPCs, but there was no evidence of its influence on the occurrence of major pulmonary complications (**grade** ≥ 3).

**Table 4:** Severity score of PPCs

Variables	Non-ONN group(n = 432)	ONN group (n = 461)	χ^2^	P-value
PPCs severity score			10.635	0.020
Grade 0 Grade 1 Grade 2 Grade 3 Grade 4 Grade 5	367(85.0) 24(5.6) 32(7.4) 8(1.9) 1(0.2) 0	416(90.2) 9(2.0) 31(6.7) 5(1.1) 0 0		
PPCs **grade**≥2	41(9.5)	36(7.8)	0.800	0.405
PPCs grade≥3	9(2.1)	5(1.1)	1.442	0.286

The original article [1] has been corrected.
